# Filling gaps in experiences religious understanding of people living with cancer in palliative care: a phenomenological qualitative study

**DOI:** 10.1186/s12904-023-01254-w

**Published:** 2023-09-05

**Authors:** Hellen Luiza Meireles Silva, Pedro Henrique Martins Valério, Cristiano Roque Antunes Barreira, Fernanda Maris Peria

**Affiliations:** 1 Department of Medical Images, Hematology and Clinical Oncology, School of Medicine of Ribeirão Preto, University of São Paulo, Ribeirão Preto, Brazil; 2 Rua Amadeu Amaral, 340, ap 112, Vila Seixas, Ribeirão Preto, SP Brazil; 3 School of Philosophy, Sciences and Letters of Ribeirão Preto, University of São Paulo, Ribeirão Preto, Brazil; 4 School of Physical Education and Sports of Ribeirão Preto, University of São Paulo, Ribeirão Preto, Brazil

**Keywords:** Spirituality, Religion, Phenomenology, Meaning of life, Palliative Care, Oncology

## Abstract

**Background:**

According to a phenomenology of contemporary religion, the analysis of religious experiences finds that they are part of an individual’s search for something powerful that overcomes him seeking not only a need, but the meaning of all existence. The present study aims to contribute to a deeper understanding of the religious experiences of people living with cancer in palliative care (PC) and fill gaps in access to experience, with regard to how it was properly lived.

**Methods:**

A qualitative, phenomenological, cross-sectional study was conducted with 14 people living with cancer undergoing PC at two outpatient clinics of a public hospital. The experiences were accessed through in-depth interviews and the results were analysed according to the principles of classical phenomenology.

**Results:**

The patients confidently surrendered to the divine, attributing to it the power of continuity of life or not, which sustained them and launched them into horizons of hope, directing them to possibilities of achieving meaning in life, which it fed back their faith and to continue living, opening them up to an intense perception of the value of life.

**Conclusions:**

The religious positions of confident surrender to the divine, to his will and a belief in his intervention, regardless of the outcome, opened possibilities to patients for the belief in the continuity of life by the power of faith. This position allowed the patients in this study to visualize achievements in the present and in the future, opening a horizon of hope, meaning and value of living. This study showed how this elements are presented and sustained, providing subsidies to health professionals seeking to provide more holistic care.

## Introduction

According to a phenomenology of contemporary religion [1], the analysis of religious experiences finds that they are part of an individual’s search for something powerful different oneself that overcomes him, with whose contact both unlimited nature this power and the limit of the human are shown. Having this awareness of the limit, this perspective philosophical outlines that the person positions himself seeking not only a need, but the meaning of all existence. Supreme power is not achieved by human beings, but it reveals itself in a way that humans have never experienced, and the human attitude towards this power is one of astonishment and, successively, of faith [2]. Several philosophical and psychological lines investigate the human search for meaning, and logotherapy and existential analysis (LAE) is one of them [3]. The logotherapeutic anthropological view understands that man is a being with psychophysical dimensions but also a noetic dimension, indicating the opening of human beings to the world [4]. This allows individuals to direct themselves to something or someone; that is, they are self-transcending beings in search of meaning in their lives and the ultimate meaning of existence [4, 5]. In this way, one enters the field of faith/theology, a field distinct from LAE, with no pretense of explaining or reducing that field to defense phychological mechanisms, for example, or the others elements belongings to psychological dimension, but accepting it in its anthropological possibility [6]. The dimension that Frankl calls spiritual or noetic cannot be confused with religiosity because the spiritual dimension is something constitutive of every human being, an expression of art, love, the encounter with the meaning of life, and relates to a Supreme Being [7].

Frankl [8] considers that the search for the meaning of the whole as well as the meaning of the conditionalities that can be affected (e.g., a disease) is the search for understanding by ultimate meaning, whereby its access can only be achieved through the domain of faith, not by human reason. The search to guide life, in line with this ultimate meaning of life or with the fundamental values or with something beyond oneself (transcendence), gives rise to a path of spirituality [9, 10]. Thus, it leans towards questions about meaning and about ultimate meaning for “someone”, who is life or God [11].

Considering the field of oncology and palliative care (PC), patients in this condition are faced with their limits, suffering and awareness of their own finitude; and may have an attitude of connection with something beyond themselves, aiming to leave authentic marks in their history and find the meaning of life [9]. The worsening of cancer disease can intensify this search for meaning [12] and find it can bring answers to the existential questions as well as well-being, comfort, hope and health [9]. Qualitative studies with people living with cancer undergoing PC are an important methodological way to describe and deepen the way in which this spiritual/religious phenomenon is experienced [13, 14]. A study by Renz et al. [15] reported that patients felt protected by the divine and that when they were suffering, they felt/saw the divine becoming present, leaving them free from fear, feeling peace due to this presence. Such experiences were not related to the end of life.

The study by Araújo et al. [14] points out that the belief in the divine sustains hope, optimism and positive expectations and offers new meanings to life and death with an influence on the overall quality of life of patients with advanced cancer. In the study by Maiko et al. [16], the patients had sense of a personal relationship with God was an important part of coping and experienced their relationship with him and the faith community as a source of strength and support, and for those who were not religious, the spirituality was the relationships with family and friends that were a source of a sense of belonging life. Some participants this study expressed feeling abandoned by God, family or faith community or had their faith shaken when wondered that God allowed this illness to occur. But other participants still felt safe by the belief that God was in control of all. Lee and Ramaswamy [17] found the search for the divine for emotional support in the experiences of illness and that conversations with God were common among patients, by which they were aided in sleep and pain management and felt calm due to this relationship with the divine.

Vasconcelos et al. [18] found reports of spiritual experiences of comfort and security in what was to come, as they trusted in the divine; attributing to him the acquisition of care, strength and patience, as well as meaning for the suffering of becoming ill, to death and the afterlife. The study by Benites, Neme and Santos [19] reports experiences of faith in cure or hope of improved health, with traces of trust in and support from the divine, promoting a meaning of life and a re-signification of family relationships, with a desire to live longer with them. There was also the appreciation and revaluation of life itself and the finding of meaning in death. Penman [20] found that patients reported how faith in God/a higher power, such as religious participation, helped them overcome suffering and relieved their depression, with the divine being allowing their hope. Voetman et al. [21] found that patients had a spiritual need to talk about death, to live their daily life without putting weight on their disease. This awareness of death caused them to have another relationship with time, establishing the priorities in their lives, but they also experienced a dilemma as to who should initiate conversations about spiritual needs. The study also pointed out how health professionals’ verbalizations about the desire for comprehensive care and nonverbal expressions, such as physical proximity and touching a hand, for example, helped patients share deeper questions related to their spiritual needs. Two recent metasyntheses, one on the experiences of patients with advanced cancer and the other on the subject of spirituality in this condition, showed different results. The first metasynthesis [22] of 23 studies and 322 patients from 11 countries, almost half of them from European countries, analysed the experiences of patients with advanced cancer but found that spirituality did not play a significant role in most studies. However, there was a relationship between religion and coping with mortality and the traumatic effects of cancer, as well as in finding meaning in life. The second metasynthesis [23] of 37 studies and 1046 participants from 18 countries, almost half of them from Asian countries, explored relevant reports of spirituality in the experience of patients with advanced cancer. This study found a significant influence of spirituality on how they experienced their illness, which helped them move from suffering to a sense of meaning, purpose and identity, allowing them to continue living despite advanced cancer.

Although they are aware of their prognoses, many patients in PC expect an improvement of their condition. The sense of hope for patients is related to their interest in aspects that are usually so important that it is not possible to live with the idea of no longer being able to reach them; thus, they are focused on their importance and not necessarily their actual reach [24]. Such elements are related to complete or future recovery, sufficient improvement to return to small, previous activities, the prolongation of life due to the advancement of medicine, the quality of life, and peace in death, which help patients to be active in life and engage in the present, helping the resilience process [24, 25]. During this stage of the disease, there may be changes in the focus on hope; when health is more favourable, hope may be focused on healing or returning to the way one was before the disease, but with a deterioration in health, hope may be more focused on making short-term plans and, for some, there is also hopelessness regarding the realization of their plans [17]. Larajeira et al. [26] showed that patients maintained hope for good results for their disease with the help of faith, even with a one-month prognosis, and focused on their quality of life, not life extension. They also pointed out that faith is a facilitator of hope, and its obstacle is the anguish felt by patients. A positioning of hope in extreme situations can help to look to the future with possibilities of fulfillment, since it is not yet known what will happen in the next moments of life [27]. Generally, hope in palliative care does not offer evidence of denial or “false” hope because patients are aware that their hope in some aspects may differ from their medical prognosis [24]. Faith can be one of these sources of hope, prolonging life through divine healing, even when aware of one’s prognosis [19, 28–30], or by just trusting in the divine will and its care [31]. Surrendering one’s situation to God has positive effects on the reduction in anxiety [28] and acceptance of illness, alleviating struggles [32].

Usually, health professionals who are faced with this type of hopeful position by spiritual beliefs feel uncomfortable because, in their view, such hope is in contrast with the fact that the disease is advanced and will likely lead to death, being this type of position, considered by professionals, a failure in communication between doctor and patient or indicative that the patient would be in denial [24, 29, 33]. Rather than relying on hypotheses and assumptions about this complex subject in a difficult context of advanced disease, the phenomenological approach helps previous concepts and theories, such as assumptions and presumptions of the researcher, give way to the experiences of the subjects who live them, considering in the investigation their characteristic elements [34]. Despite advances in the qualitative studies field of palliative care in oncology, some studies present possible methodological issues that can be advanced and gaps can be filled. Among that issues, it can be highlighted the unquestioned influence of theoretical assumptions in interview scripts inclining participant’s statements to rationalize under the researcher’s terms; a focus on the disease, on effects of coping without a more comprehensive description of how a person’s experience is affected; and, finally, deficiency in the in-depth descriptions of essential elements of how people live their religiosity and how, under the condition of advanced disease, they position themselves, living an attitude to be focused through a comprehensive understanding of their lived experience, especially how their positioning is sustained. In addition, qualitative studies are indicated in the field of PC to understand in depth the experiences of patients, which may assist healthcare providers in delivering the type of comprehensive care that the area requests [13, 35, 36]. Therefore, the present study aims to contribute to a deeper understanding of the religious experiences of people living with cancer in PC by and fill gaps in access to experience, with regard to how it was properly lived, unlike theories and objectifications about it, so that therapies can be traced closer to the context experienced by the patient and approximations between the patient´s expectations of cure or improvement and the professionals´perpsectives can be made.

## Method

### Study design

An excerpt from the study by Silva, Valério, Barreira, Peria [36] was provided to analyze the religious/faith experiences of the participants, as they emphasized them in their experience reports. The original study investigated the essential elements of the personal positioning of patients with advanced cancer and how they support each other. It is worth noting that the religious experiences of these patients were not the subject of the study and were not included in the interview guide but appeared spontaneously in the experience reports of all interviewees, justifying the relevance of the present investigative focus. The study was cross-sectional, and the theoretical framework was based on the LAE and classical phenomenology of Husserl and Edith Stein. The Consolidated Criteria for Reporting Qualitative Research [37] were applied in the assessment of study quality.

### Participants and procedures

In the present study participated 14 people living with cancer in PC, with good performance (Karnofsky Performance Scale [38] greater that 70) and that were outpatient in follow-up care at the Clinical Oncology Services and/or Palliative Care Group of Hospital das Clínicas of School of Medicine of Ribeirão Preto of the University of São Paulo (Onco-GPC-HC-SMRP-USP). The religious affiliations of the participants included 7 catholics, 4 evangelicals, 1 with spirituality (believe in God), and 2 spiritists (believe in reincarnation). This fact occurred by chance and was part of questionnaire to collect sociodemographic data. The limit of this sampling was based on data saturation according to this type of qualitative phenomenological approach. In the present study, the classical phenomenological method applied in research in psychology was used in all phases. Both the phenomenological operation of suspensive listening (putting preconceptions of the object in parentheses) and empathic listening (experiencing the experience of others facilitating focus on the object and understanding of the reports) cause the interlocutor to produce very descriptive reports focused on the object of study [34]. Thus, these reports offer much detail, depth and volume of information without requiring a large number of people [39]. Hence, the variable possibilities of describing the phenomenon began to be exhausted, and the invariable experiences became present, leading to data saturation [40]. Two other aspects present in this study that allowed the quality of the results and the consequent data saturation are as follows: the patients chosen for the study had experience relevant to the study question, since they were aware that they were in palliative care, and the first author had experience in the area of palliative care research and as a psychologist. The second and third authors had expertise in conducting phenomenological interviews and supervised their performance. These aspects are important criteria for data saturation because they facilitate asking questions that lead patients to delve deeper into their important experiences related to the object under study [39]. In view of these aspects, it was possible to determine data saturation and interrupt data collection Patients who were treated by the first author, a psychologist at the clinic, were not included in the study. The inclusion criteria were as follows: patients older than 18 years of age; both sexes; with a diagnosis of malignancy confirmed by anatomopathological examination (for malignant neoplasia of the hepatocarcinoma type, diagnosis was considered according to the specific criteria of this neoplasia); who were aware that their illness was in the palliative stage; of any location as the primary site; with IV being updated at the time of recruitment for this cancer study; with Karnofsky Performance Scale (KPS) [38] greater than or equal to 50 and in outpatient clinical follow-up by Onco-GPC-HC-SMRP-USP. The exclusion criteria were as follows: people living with cancer undergoing curative cancer treatment, patients with noncancer diseases and those who were not treated by the first author. The exclusion criteria were patients with difficulties in understanding the free and informed consent form (ICF) and the questions contained in the instruments and in the interview (2 patients).

Patients were initially screened from the general list of patients scheduled daily, and after consulting their medical records, the inclusion and exclusion criteria were checked. Next, the patients that were within the inclusion criteria were invited to participate in the study in the outpatient clinic waiting room. Those who agreed to participate were taken to the PC room where the instruments were administered. Before data collection, the 14 participants read and signed the ICF After data collection, the last author, a medical oncologist, verified and confirmed that all patients were in the palliative stage. A questionnaire was also performed to collect sociodemographic data and clinical information contained in the medical records. The time of the interview was approximately 45 min.

### Data collection

The phenomenological interview was chosen because it is a means appropriate to the object of study, allowing patients to expressively narrate their experiences under the conduction of suspensive listening, which allows access to the way patients experienced the phenomenon. The participants were invited to give a spontaneous and detailed account of their experience to enable first access to the experiences. The objective was not to obtain rationalizations about the experience, but to generate an account of what it was like to experience it, to know how it occurred to the consciousness of the participant and the researcher. The reports of experiences are significant moments in the phenomenological interview because it achieves this goal [34].

The in-depth semi structured interview, developed by Silva, Valério, Barreira, Peria [27], began with a contextualization question that aided in the understanding of the biography and the personal context in which the experiences gained meaning [41]. The interview guide sought to faithfully explore the object of experience, according to the phenomenological reduction prior to the empirical phase [34, 42]. The starting point was the question “How did your illness begin?” The other guiding questions were asked in a logical sequence and grading the complexity and specificity of the object in order to allow the interviewer to deepen his/her experiences [41]. The guiding questions were: “How is it for you to go through the illness experience?”, “How are you dealing with this illness experience?” Based on the responses, other questions were asked gradually to guide the thematic experience (position regarding illness), elucidate and deepen it. To achieve this objective, empathic monitoring was necessary, which is an attitude and synthesis of intersubjective operations guided by empathy to accompany the reported experience and clarify it, when necessary, seeking its meanings and lived experiences [34]. The interview was conducted by the first author and supervised by the second and third authors, who are experts conducting in this type of interview, which helped remove any preconceptions and theories about the object.

### Data analysis

For the performance of the phenomenological interview, a series of operations were performed to let the lived experience of the participant fill the theme prompted by each question [34]. The interviews were recorded with the consent of the participants and fully transcribed by the researcher for later analysis.

The qualitative data were analyzed according to Barreira and Ranieri [34]. The first step was psychological reduction, i.e., the researcher captured and described significant moments related to the researched object and the connections between them in each interview. The significant moments are expressions of the lived experience in the personal attitude (participant describes how the experience was lived and not facts or rational information about it). Then, an intentional crossing was performed to take all the variations of the lived experiences and seek in them what was common, excluding accessory characteristics, to obtain the essential aspects that make the phenomenon to be recognized as such.

### Ethical aspects

All ethical principles in research involving human beings in Brazil were considered as well as the Declaration of Helsinki. The study was approved by the research ethics committee of the institution where it was conducted by Process No. 10,402/2017.

The ICF was signed by the participant and the researcher in two copies, one of which remained with the participant.

## Results

All patients interviewed had a religion or spirituality and there were no atheists or agnostics in this sample (which occurred by chance).

### Surrender to the divine will

The experience of the relationship with the divine covered several aspects of the illness experience:

“*So, well… I don’t know how I’ll end up. (…) So, I said: “Look, I’m going to put myself in God’s hands”. I’m Catholic, okay? So I say, “His will be done.“ Right? (…). So, I just ask God to give me quality of life, and I’m having it.”* (Interviewee 1).

On the one hand, faced with the uncertainty of future events related to treatment and death – “*I don’t know what my end will be like*”, the patient turns to surrender to the divine – *so I said: “Look, I’m going to put myself in God’s hands”.* Such surrender reveals itself as a kind of acceptance or desire that *“his will be done”*, which, on the other hand, opens the possibility for requests to be made of him: if they are met, his will is being done - *I only ask God to give me quality of life, and I’m having it*. However, if there is a worsening or an event that is unpredictable to the patient - which, therefore, is outside the horizon of their requests because it already presupposes the desired object to come as a possibility known in advance - these are also perceived by them as the fulfillment of the will of God. That is, surrender is motivated by the uncertainty of future events: *I do not know how it will turn out, so I will put myself in God’s hands*. *Thus*, *putting oneself in God’s hands* appears as a way of accepting one’s own not knowing what is going to happen, of one’s own uncertainty that, for the patient, is now always the manifestation of God’s will. Otherwise, surrendering to the divine has the same meaning as surrendering to events that are beyond the patient’s control, but because they are now the very manifestation of the divine will, they can be perceived as the manifestation of God’s action in their lives.

However, this surrender is different from a pure and simple surrender to unpredictable events or not: it is a surrender to the divine will - and correlatively to the events of life, in order to take an active part and act within its possibilities - that have a meaning that is confirmed, especially when events are favorable to the patient and his improvement:

“*(…) I had surgery and left here wonderful, very well. Nobody said I had surgery. (…). So I say: “He is with me”. I’m happy about that, because in everything I see Him giving me that support, that strength (…). So, I trusted a lot and I am so far… confident, I want to live, do you understand? If it’s with His permission and I’m sure He wants it too.”* (Interviewee 2).

The “presence” or “performance” of the divine is also perceived by patients through the events that benefit them: *I had surgery and left here wonderful*, *so I say: “He is with me”.* What, from a skeptical perspective, would be a coincidence between believing and asking with the advent of a successful surgery - *I left here amazed* - is perceived, from the patient’s perspective, as the presence or caregiving intervention of God: care divine reveals itself as the fulfillment of events, through belief and surrender (especially when the desired outcome coincides to some level with the factual outcome), with the sense of the presence and action of the divine. If unexpected events, even if unfavorable, are in the same way, then the meaning of God’s presence is revealed in the filling of uncertainty itself, of the unexpected itself with the meaning of his will - *If it’s with His permission and I’m sure He wants it too.* More precisely, the meaning of events and the meaning of the will of God, for those who believe in him, begin to manifest themselves in one and the same sense. Since the relationship between asking for something – wanting to live –, trusting in, and the event to come, positive in the realization of the latter – *because in everything I see Him giving me that support, that strength* – comes the experience of obtaining strength, care, as the manifestation of their will. This happens, especially when it coincides with the will of God, in this perspective: *So, I trusted a lot and I am so far… confident, I want to live, do you understand? If it’s with His permission and I’m sure He wants it too.*

### Divine intervention and the meaning of events

In this relationship between patients and the divine, an experience of confident surrender of their disease and their future to the divine appeared: within this surrender, the events of improvement or worsening of their condition or treatment are perceived by them as forms of manifestation of divine will and, correspondingly, of his presence and care. It is possible to say, for now, that the manifestation of the divine will is revealed as the manifestation of an act of giving meaning to events that, in themselves, have no other meaning than what, objectively and factually, they cause by chance, improvement or worsening and, ultimately, the certainty of death. Given the proximity of this as a certainty or more likely end, the belief in miracles appears, resulting in an experience of comfort, although this time without being accompanied by requests, giving even more extension to the uncertainty filled with the will of God, in which healing. It is only performed if he deems it appropriate:

“*Look… I get emotional. We are Catholics and very religious, so we believe in miracles. So, prayer brings comfort (…). We pray normally, we don’t keep asking: ‘Oh, heal me, heal me’, not at all. Healing can come if God sees fit.”* (Interviewee 10).

The meaning of the divine help, presence and action becomes, therefore, inseparable from the meaning of the events themselves, which becomes more complex according to the narratives of each religion. In this sense, adhering to treatment – one among other factual events in the world – comes to have the same meaning as such orientation to the divine, and is also a possible form of relationship, belief or surrender to it:

*“He is helping me a lot. I am His child, am I not? So, I am His son and I pray a lot for Him to help me and… so, I think that on my way today until the end, what I see at the end of the tunnel is a total control of the disease since I Always be comfortable with the treatment when necessary, understand?”* (Interviewee 14).

### Religious faith in the continuity of life and the opening of horizons of achievements

The way patients attribute meaning and surrender is also revealed as a modality of self-projection in the future:

“*(…) it is a situation that God will give me 14 more years, so I can celebrate my golden wedding anniversary with my husband yet, so there are 14 years to go.”* (Interviewee 1).

“*I think that if I am alive, I am only alive because of faith. People despair and despair is worse, right? You get worried and being worried all the time is worse (…). I think that faith helps one not to despair anymore, better put it this way, to have hope, to have hope for the future, to live longer (…).”* (Interviewee 10).

*“(…) I thank God very much because it is faith that I have that helps a lot, right? And I go on with my life… (…) (crying). It helps because I have faith that everything works out for me.”* (Interviewee 12).

The faith/belief that they will not die soon appeared in all the patients’ experience reports, i.e., the belief in the continuity of life in this condition of incurable illness, with aspects of security and hope, launching them to a horizon of hope possibilities of achievement in the present and in the future. In this sample, faith/belief was closely linked to religious faith, in which the continuity of life is attributed to the divine will. Simultaneously and correlatively, such emphasis on the continuity of life presupposes, in the affective constitution of its horizon, both the factual and concrete dimension of the illness and its consequences and, and especially, the resulting psychic suffering. Thus, ultimately, the finitude of this life that one wants to perpetuate with this same faith, takes part in the constitution of the meaning of the motivation of the latter, which also appears as an avoidance of overestimating such finitude and the factual consequences of falling ill: *I am only alive because of faith. If we despair, it is worse. You get worried and being worried all the time is worse.* Thus, faith has a double potentiation of positive engagement in life, in its here and now and in its possibilities, as in the prolongation of life toward the future: *faith helps not to despair anymore*– it helps to accomplish what is achievable, in the here now of life, it would be withdrawn or impaired by despair – and, correlatively, *to have hope for the future, to live longer.*

This experience of engagement, through faith, in the continuity of life, both in its here and now, and in its desired future, is also updated and reinforced in the attentive and affectionate presence of the medical team and close people who, together with the patient, do not emphasize the illness, which remains factual and pre-thematic or pre-reflectively present: *so I do not even think*, *it does not even cross my mind* : *“Wow, I have a tumor”.* However, such departure from the factual conditions of illness allows for an intersubjective emphasis on the potential and possible dimensions of life, that is, the treatment itself aimed at improving the health conditions of patients:

*“I made a lot of friends with a doctor, nurse, I get there to do the chemo. Here it comes, X (name omitted), comes in I don’t know who is our lady! So I don’t even think about it. I’m there doing the chemo, taking the serum, that medicine and X (name withheld) said: “Look at the white woman”. “That’s the one I really like.” Taking it like this, it doesn’t even cross my mind: “Wow, I have a tumor.”* (Interviewee 2).

*“Without help, we can’t win, from the doctors, from friends, from the family. I have a pastoral meeting, and I have a meeting of the other pastoral, people say: “Look, we put you in prayer yesterday (…)”. So, all I hear is this… and I pray too… ah, I’ll get far, God willing.”* (Interviewee 9).

### Desires and reasons to live to the fullest

Faith is both reinforced and deepened, as it reinforces and deepens, intersubjectively and socially. The potentiation of life in its here and now, in its practical and existential possibilities, as in its possible future, as hope: it interweaves different social and medical experiences in the world, as effective as possible, towards living more in general, treatment being an integral part of this direction. The most diverse reasons why patients want to live appear in this living, directing and propelling them toward present and future horizon:

*“(…) I want to see my grandson graduated (…) so I keep that hope, of all the good that I wil still live, you know?!” (Interviewee 9)*.

“*(…) I love life (starts to cry) (…). My emotion is not as you say with despair, it is joy, it is thanksgiving, for faith, for understanding… (…) I like to go to church, sing, pray, I am a minister of the Eucharist too (…)”.* (Interviewee 10)

Patients reported people and activities that were significant in their lives and wanted to direct themselves to live them as well as live new achievements in the future. Thus, meaning in life and its value appeared to the patients, having a desire to live intensely.

Therefore, in short, the intentional structuring of the meaning of faith is shown as an incessant – sometimes hesitant – reconduction, both reflective and pre-reflective, of lived experiences in the world, based on what, in them, life shows as a possibility and potential, both already completed and in the future, of living intensely. Adhere to medical treatment is shown, in this experiential context interwoven by faith, as a part, both reconductive and reconducted, by filling the meaning of the lived events with the meaning of the fulfillment of the will constitutive of the experience of faith. Since the latter is directed at life, everything in the patient’s concrete life that can be interpreted as a reaffirmation of their potential and possibilities becomes a field of engagement of their actions, toward the continuity of life. without, however, conflict with the acceptance of the advent of finitude or its approximation, because even these, appear there filled with the meaning of the fulfillment of the will of the divine.

## Discussion

The person religious can understand many issues of his/her life and the meaning of his/her suffering, realizing that there is nothing in vain in his/her life, nothing meaningless for him/her, even in the face of tragedy [27]. In this study, amid the unstable situation of advanced cancer, participants report confident surrender of their lives to the divine, to his will and a belief in his intervention, regardless of the outcome. In addition, it was identified in the patients’ experience that they felt cared for and safe in the relationship with the divine. This may facilitate the acceptance and coping with this condition of advanced cancer [18, 19, 32, 43].

There was, therefore, the opening of possibilities for the belief in the continuity of life by the power of faith, even with advanced cancer, which allowed the patients in this study to visualize achievements in the present and in the future, thus opening a horizon of hope, adherence to a power of continuity readily threatened by finitude. Others studies confirmed this belief in the continuity of life by divine intervention [19, 28–30] The glimpse of total or absolute power is contemporary with the realization of impotence by human beings who have evidence of their limits [1, 2, 44]. This inauguration of the religious experience can unfold in all forms of religious practice, such as investment in faith as a search for overcoming one’s own impotence and adherence to divine power [1]. This opens the way to the theme of hope that is a frequent theme among patients undergoing PC, notably the hope of a cure, highlighted in many studies, whether or not it is related to religiosity [24, 25, 28, 29, 45]. Hope is considered a central part of the resilience process due to the strengthening of the attitude oriented toward life in the face of the decline announced by the disease [32], crossing the religious or spiritual meaning for people who believe or adhere to it. This shows the strength and therapeutic potential of experiencing hope, an important warning that contradicts a common interpretation in health care: that hope is a denial of reality [24, 29, 33]. The experience of hope in the continuity of life also materialized in the attentive and affectionate presence of the medical team and close people who, together with the patient with advanced cancer, enhanced the possibilities of living. Robinson [24], in his study on hope in this context, noted that longer consultations performed by the same doctor were experienced by patients as an investment in their health status and not giving up on them because they were on PC. This gave them greater motivation to put their hope into practice in the search for the meaning of life. These data support and may motivate the rethinking of PC care models, especially when enriched by the results of this study, which demonstrate that there is an articulation between the motivation toward the future of a life that continues and the intensification of the potentialities and possibilities of its life lived here and now. From the phenomenological perspective, this is the original “place” of any experience and, ultimately, of time itself.

The will to live was an element that values life to the fullest and provides support for reasons to live. Patients in this context cling to the life they want to live with all their intensity [13, 22]. Patients in the present study had people and activities that were significant to them and were directed toward living these experiences in the present and future, thus appearing the meaning and value of living. This element of the meaning of life is rarely found in the literature and has not yet been articulated with regards to the hope for continuity of life through religious faith. Such data may contribute to the understanding of the nuances of patients’ religious experiences and help them find possible reasons for living that can sustain their hope and strength to continue dealing with their disease and finitude. This position is similar to the vision of person and the world presented by psychiatrist Viktor Frankl, who pointed to the search of human beings for the meaning of their lives and its unconditional value “until their last breath” [4]. Even in a complex context permeated by the challenges of living with an incurable cancer, it is possible to find meaning in daily life and affirm life [43]. The author’s therapeutic approach, LAE, has been applied and indicated for patients undergoing PC [43] and, more specifically, for spiritual care in this context, as it helps in the attitude of seeking meaning in suffering [46].

The patients in the present study believed that divine action prolongs their lives in this material and earthly realm and in the present time, but not only: such experience of faith reveals itself as a way of reconfiguration of the horizons of lived experiences of each here and now and its possible futures and correlates, intensifying the importance, desire and value of living more in the present and future. In turn, studies by Arrieira et al. [47], Benites, Neme and Santos [19] and Vasconcelos et al. [18] showed that the patients believed in the continuity of life after death. These studies do not accurately describe the severity of the illness at the time of the interview, but due to the type of care, one performed at home and the last two in the ward, the disease could have been more aggravated. One hypothesis would be that the patients in the present study, because they were in outpatient care and with good functionality (KPS greater than 70), experienced their religiosity without thematizing death, and those who were more seriously ill could experience finitude more intensely and their spirituality would cross such experience as support, conceiving the continuity of life after their death. Both conceptions of hope in the present or after death are encompassed in Christian hope – which is the majority of study subjects and the Brazilian population which [48] points to the concomitant of this hope in this world and to divine communion in heaven [49]. More research is needed to understand this difference and its possible implications for patient experience and healthcare interventions.

When interpreting the meaning of the patients’ religiosity as the search for a “magic cure” to control the uncontrollable situation of the disease, clinging to a “supposed power of faith”, the study by Reis, Farias and Quintana [29] diverges so much from the present study and the Brazilian and international literature mentioned above. The result indicated by the authors describes that the patients understood that the divine would be responsible for the continuity of life, surpassing the scientific method of treatment, being an illusory control and that, although religion offered positive psychic effects, the patients showed insecurity and desperately wanted healing, thus considering faith as a “defense that fails” and a contradiction. In contrast, in view of the present study and the current literature, it was identified that people living with cancer in PC, when experiencing their spirituality, were not immune to negative feelings or internal conflicts related to the disease but replaced themselves in a way engaged in reality and in treatment, which helped them maintain the positioning of hope in continuing to live life as long as possible [18, 19, 24, 25, 28, 32] as well as relieved them of psychological symptoms, such as depression [20, 22, 47]. The study by Arantzamendi et al. [32] helps in understanding the phenomenon of this type of illness, pointing out that patients with advanced oncological disease put the disease and its negative emotional impact in the background and put living life in the first place. significantly possible through various attitudes and efforts, including faith, in which the experience of a trusting relationship mitigates the negative impact of the disease. Therefore, it can be understood that the study by Reis, Faria and Quintana [29] points out more to the permanence of the patient’s suffering despite religious adherence than, as presented here, the psychic and existential movement that unfolds along with the experiences of faith. Considering the structure of religious experience and its broader anthropological dimension, it is impossible to reduce it to a defense mechanism, although forms of distance from the latent despair in the face of finitude. For example, take part in its constitution, however, it is important to affirm that such departure from factuality as a source of despair occurs precisely in favor of what, in this very factuality, is potency, concrete possibility, as well as hope. If the study by Reis, Farias and Quintana [29] also denied religious experience when they pointed out that patients clung to the “supposed power of faith” and “in an illusory way” (p. 112–113) from the perspective of this study, when the judgment regarding the factuality of such power is suspended in the face of the objective physical and biological variables, the structuring of the meaning of their experience is revealed as a redirection of the patients’ motivations toward the optimization of treatment and of the actual life itself in general, in its way of being lived.

Finally, the results and reflections of a phenomenological nature of the present study bring a novelty to the discussion, because this method puts in parenthesis interpretations and assumptions to allow the possibility of returning to the things themselves, as the phenomenon shows itself to consciousness [34], reducing the risk of hasty and theoretically predetermined interpretations. In the field of psychology, the LAE of Viktor Frankl has also warned, since the last century, about the danger of reductionism, psychologisms and, warned of the importance of considering, in investigations in psychology and psychiatry, what is specific to the human being, or that is, its noetic dimension and, thus, enable truly humanized care [5, 6, 8].

The limitations of the study are that there was no diversity of religious (the Cristian majority) and there were no atheists or agnostics in our sample, which could modify or expand the results regarding faith in the continuity of life or the way of dealing with illness and death. Therefore, further studies that include these populations are needed.

## Conclusion

The experiences of the patients in the present study have revealed a way of attributing divine meaning to the condition of advanced cancer, decisively modifying not only how they understand but also how they engage in life. This attribution of meaning occurs in the modality of a surrender to what is independent of the will of the patients and affects them. This type of religious experience opens horizons of possibilities of continuation and fulfillment in life for these patients through the experiences of hope, extending their time to the maximum, even if in the short term and in an indeterminate way. At the same time, through their engagement in each here and now of life, considerations of their reasons for living, their value of life and their use of the possibilities that arise emerge in the lives these patients. These results, obtained by the phenomenological approach, have elucidated how these elements are present and sustained, providing implications for health professionals who seek to offer more comprehensive care to their patients.

## Data Availability

The qualitative datasets generated and/or analyzed in the study are not publicly available due to the need to protect patients’ identities but are available from the corresponding author on reasonable request.

## List of abbreviations

LAE: Logotherapy and existential analysis
PC: Palliative care
Onco-GPC-HC-SMRP-USP: Clinical Oncology Services and/or Palliative Care Group of Hospital das Clínicas of School of Medicine of Ribeirão Preto of the University of São Paulo
KPS: Karnofsky Performance Scale
ICF: Informed consent form
